# Clinical Heterogeneity of Duchenne Muscular Dystrophy (DMD): Definition of Sub-Phenotypes and Predictive Criteria by Long-Term Follow-Up

**DOI:** 10.1371/journal.pone.0004347

**Published:** 2009-02-05

**Authors:** Isabelle Desguerre, Christo Christov, Michele Mayer, Reinhard Zeller, Henri-Marc Becane, Sylvie Bastuji-Garin, France Leturcq, Catherine Chiron, Jamel Chelly, Romain K. Gherardi

**Affiliations:** 1 Department of Neuropediatrics, Neuromuscular Disease Reference Center “Garches-Necker-Mondor-Hendaye”, Necker - Enfants Malades Hospital, Paris, France; 2 Department of Neurosciences, Team 10 INSERM U841 Mondor Biomedical Research Institute, Paris XII University, Créteil, France; 3 Cellular and Tissular Imaging Plateform, INSERM U841 Mondor Biomedical Research Institute, Créteil, France; 4 Department of Neuropediatrics, Trousseau Hospital, Paris, France; 5 Department of Biochemistry and Genetics, Cochin-Saint Vincent-de-Paul Hospital Group, Paris, France; 6 CNRS (UMR 8104), Institut Cochin, Université Paris Descartes, Paris, France; 7 Public Health and Statistics Department Henri Mondor Hospital, Créteil, France; 8 INSERM U663, Paris V University, Paris, France; Stanford University, United States of America

## Abstract

**Background:**

To explore clinical heterogeneity of Duchenne muscular dystrophy (DMD), viewed as a major obstacle to the interpretation of therapeutic trials

**Methodology/Principal Findings:**

A retrospective single institution long-term follow-up study was carried out in DMD patients with both complete lack of muscle dystrophin and genotyping. An exploratory series (series 1) was used to assess phenotypic heterogeneity and to identify early criteria predicting future outcome; it included 75 consecutive steroid-free patients, longitudinally evaluated for motor, respiratory, cardiac and cognitive functions (median follow-up: 10.5 yrs). A validation series (series 2) was used to test robustness of the selected predictive criteria; it included 34 more routinely evaluated patients (age>12 yrs). Multivariate analysis of series 1 classified 70/75 patients into 4 clusters with distinctive intellectual and motor outcomes: A (early infantile DMD, 20%): severe intellectual and motor outcomes; B (classical DMD, 28%): intermediate intellectual and poor motor outcome; C (moderate pure motor DMD, 22%): normal intelligence and delayed motor impairment; and D (severe pure motor DMD, 30%): normal intelligence and poor motor outcome. Group A patients had the most severe respiratory and cardiac involvement. Frequency of mutations upstream to exon 30 increased from group A to D, but genotype/phenotype correlations were restricted to cognition (IQ>71: OR 7.7, 95%CI 1.6–20.4, p<0.003). Diagnostic accuracy tests showed that combination of “clinical onset <2 yrs” with “mental retardation” reliably assigned patients to group A (sensitivity 0.93, specificity 0.98). Combination of “lower limb MMT score>6 at 8 yrs” with “normal or borderline mental status” reliably assigned patients to group C (sensitivity: 1, specificity: 0.94). These criteria were also predictive of “early infantile DMD” and “moderate pure motor DMD” in series 2.

**Conclusions/Significance:**

DMD can be divided into 4 sub-phenotypes differing by severity of muscle and brain dysfunction. Simple early criteria can be used to include patients with similar outcomes in future therapeutic trials.

## Introduction

Affecting approximately one in 3500 males, Duchenne muscular dystrophy (DMD) is the most common inherited myopathy whose poor prognosis is well known [1]. DMD has been classically considered stereotyped in its clinical presentation, evolution and severity [2], [3]. However, inter-individual differences in terms of motor, respiratory and cardiac involvement had been reported before the identification of dystrophin [1]. Subsequently, rare studies documented that identical mutations can produce DMD phenotypes of different severity [4]. Adding to this clinical heterogeneity, brain dysfunction is observed in some DMD patients [5]. As recent preclinical studies have opened avenues for promising pharmacologic, gene and cell therapies of the disease [6], in-depth knowledge of DMD natural history is now mandatory. In fact, phenotypic variations were already shown to compromise results of clinical trials [7].

In the literature, large DMD series investigated on a follow-up basis are scant, out-dated or fragmentary. The largest study, analyzing the history of 473 Dutch DMD patients diagnosed from 1961 to 1982, provides limited information, since neither muscle biopsy nor genetic analysis was available for most cases, data was collected by a questionnaire sent to physicians, and clinical practices were not described [8]. Studies searching for genotype-phenotype correlations considered the whole spectrum of dystrophinopathies and not homogenous DMD cohorts [9], [10]. Expectedly, inverse correlation was found between severity of disease and residual amount of dystrophin assessed by immunoblotting [10]. Studies comparing muscle testing scores in DMD patients are mostly cross-sectional [11]–[13]. Longitudinal evaluation has been limited to respiratory and cardiac functions studies which demonstrated the beneficial effects of ventilation support [14], [15] and early angiotensin-converting enzyme inhibitor (ACEI) administration [16].

We report a comprehensive clinical analysis of 75 DMD patients (series 1), evaluated longitudinally by the same team over a mean follow-up of >10 yrs. This analysis substantiates DMD's clinical heterogeneity and identifies 4 phenotypes with different outcomes that can be predicted by simple clinical indicators. The applicability of these indicators was successfully tested in a second series of 34 patients.

## Methods

### Patients

DMD diagnosis was performed from 1990 to 2000 at Saint-Vincent-de-Paul hospital, Paris; in both series 1 and 2, inclusion criteria were: (i) absence of muscle dystrophin assessed by both immunohistochemistry (Dys1, 2, 3) and Western blot (dystrophin bands absent) (moAbs purchased from Novocastra, UK, see below); (ii) genotyping performed (laboratory of molecular genetics, Cochin hospital, Paris); (iii) follow-up >4 yrs by the same team.

Diagnosis and medical care of patients were standardized as recommanded by the French consortium on DMD management. Muscle biopsy was used to assess DMD diagnosis on the basis of histology, immunohistochemistry and Western blot. According to current rules in France, both children and parents gave written individual informed consent to participate to the clinico-genetic study and approval was obtained from the Assistance Publique-Hôpitaux de Paris (APHP) institutional ethics board (CPPRB of Cochin hospital).

### Follow-up

Children from series 1 (n = 75) were systematically evaluated every six months by a multidisciplinary team for motor, respiratory, cardiac, and nutritional status and remained steroid-free. Physiotherapy was performed 2–3 times per week. Lower-limb contractures were prevented by nocturnal leg orthesis from age 6, and ultimately treated by tenotomy. Spinal arthrodesis was systematically performed from age 12. Respiratory care started when forced vital capacity (FVC) reached 1l or 30% of the theoretical value and included nocturnal monitoring of capillary PO_2_ and PCO_2_, intermittent positive pressure ventilatory (IPPV) daily support, and nasal non invasive nocturnal ventilatory (NINV) support in case of abnormal blood gas levels. Cardiac echogram was performed yearly and myocardial scintigram immediately before surgery; ACEIs were administered when left ventricular ejection fraction (LVEF) reached 55%. Children from series 2 (n = 34) were aged more than 12 yrs at data collection and had been evaluated and taken in charge at Necker hospital, Paris, using standard procedures.

### Clinical parameters

Initial exploration yielded 33 (out of 82) relevant items describing history of disease, muscle, cardiac, respiratory and cognitive functions, and genetics (Table 1).

**Table 1:** Data describing patients' history of disease, muscle and cognitive functions (quantitative variables are mean±SD).

History	Age at initial symptoms (yrs)	Initial symptom: psychomotor delay (%)	Initial symptom: abnormal gait (%)	Initial symptom: high CK (%)	Age at diagnosis (yrs)	Age at follow-up end-point (yrs)	Age at first walking (months)	Age at loss of ambulation (yrs)	Age at becoming chair-ridden (yrs)	Age at beginning of physiotherapy (yrs)	CK level (IU)	Phenotype fat/thin/normal (%)
Cluster A N = 14	1.3±0.6	100	0	0	4.1±2.1	13.5±3.7	20.0±7.9	9.0±1.6	8.4±1.5	4.5±2.0	17765	50/29/21
Cluster B N = 19	3.6±1.7	11	68	0	5.6±1.7	15.3±3.8	16.0±4.8	9.6±1.3	9.5±1.3	6.3±1.4	8166	32/42/26
Cluster C N = 16	3.8±2.0	0	44	13	7.2±1.9	16.8±1.6	15.0±3.8	12.6±2.0	11.5±1.5	8.1±2.8	9000	12/69/19
Cluster D N = 21	3.3±1.72	0	33	19	4.5±1.9	15.7±3.4	16.0±3.4	10.0±1.4	9.0±.0	5.4±1.7	9566	29/38/33
**Overall N = 75**	**3.0±1.86**	**24**	**65**	**10**	**5.3±2.1**	**15.4±3.4**	**16.8±5.1**	**10.3±1.9**	**9.5±1.7**	**6.0±2.3**	**9680**	**32/40/28**

Muscle function	MMT 9 muscles at 8 yrs	MMT lower limbs at 8 yrs	MMT upper limbs at 8 yrs	MMT from 9 muscles at 10 yrs	MMT lower limbs at 10 yrs	MMT upper limbs at 10 yrs	Testing of quadriceps at 10 yrs	Age lower limbs contractures (yrs)	Age scoliosis (yrs)	LEFVG <55% before 10 yrs (%)	Vital capacity at 10 years (%)	Age abnormal residual volume (yrs)
Cluster A N = 14	6.5±0.7	6.7±1.5	6.3±0.9	5.5±1.0	4.8±1.0	6.1±1.1	2.1±0.4	6.3±1.4	11.6±2.0	37	65±16	8.9±1.5
Cluster B N = 19	6.1±2.4	6.2±2.5	6.0±2.5	5.8±0.8	5.3±1.2	6.3±0.6	2.4±0.5	7.7±1.5	11.9±1.3	11	75±16	9.4±2.2
Cluster C N = 16	8.0±0.7	8.0±0.7	8.0±0.8	7.0±0.6	6.8±0.6	7.2±0.7	3.1±07	10.2±2.1	13.2±1.4	0	80±14	10.9±1.3
Cluster D N = 21	6.8±0.7	7.1±0.7	6.6±1.0	5.6±0.6	5.1±0.6	6.0±0.7	2.5±04	7.1±0.3	12.4±1.5	6	81±12	10.4±2.3
**Overall N = 75**	**6.7±1.5**	**7.0±0.5**	**6.6±1.6**	**5.9±0.9**	**5.5±1.1**	**6.4±0.9**	**2.5±06**	**7.8±2.1**	**12.2±1.6**	**27**	**75±15**	**9.9±2.0**

Cognition	Severe mental retardation (%)	Moderate mental retardation (%)	Neuropsychological dysfunction (%)	Normal mental status (%)	Ordinary school (%)	Motor handicapped school (%)	Mental handicapped school (%)	School delay >3 yrs (%)	Genetics deletion/mutation/duplication (%)
Cluster A N = 15	29	57	14	0	21	43	36	86	66/33/0
Cluster B N = 23	6	42	52	0	26	63	11	26	90/5/5
Cluster C N = 16	0	0	31	69	38	62	0	6	73/26/0
Cluster D N = 21	0	0	0	100	86	14	0	0	71/19/10
**Overall N = 75**	**6**	**24**	**27**	**43**	**48**	**43**	**9**	**24**	**77/19/4**

Manual muscle testing (MMT) using the Medical Research Council (MRC) scale [14] was yearly performed, yielding upper limb (average value of 2×5 muscles), lower limb (2×4 muscles) and global (2×9 muscles) scores reported at ages 8 and 10. Respiratory function was yearly evaluated and 3 indices were derived from FVC, residual functional capacity (RFC), and residual volume (RV) expressed relative to theoretical values: (i) FVC decrease from 10 to 14 yrs (ΔFCV); (ii) RFC plateauing (age of pulmonary growth arrest); (iii) onset of RV increase (age of involvement of expiratory muscles). Cardiac function was yearly assessed and the age at decrease of LVEF below 55% was considered. Body mass index determined at age 8 was classified as low, normal, or high according to reference curves [17]. Electroretinogram was scored as normal or abnormal.

Cognitive status was evaluated by both general intelligence assessment (IQ) and educational level. Wechsler Intelligence Scale [18] was used to measure, from age 6 to 10, verbal performance and full scale IQ. Patients were conventionally classified [18] as (I) severely mentally retarded when IQ was <50 or impossible to assess because of pronounced behavioural disturbances; (II) mildly mentally retarded (50<IQ<70); (III) borderline (71<IQ<84); and (IV) normal (IQ>85 or strictly normal academic level). Were also considered school delay; the maximal education level reached by patients, and the type of establishment attended according to the French educational model (Table 1).

### Molecular and protein analyses

Muscle proteins were screened by immunihistochemistry and two multiplex Western blot analyses: (A) combination of Dys8/6C5 (NLC-DYS2/dystrophin C-ter), Cal3c/2A2 (NLC-CALP-12A2/calpain 3 exon 8), 35DAG/21B5 (NLC-γ-SARC/γ-sarcoglycan), and Ham1/7B6 (NLC-Hamlet/dysferlin) monoclonal antibodies; (B) combination of Dys4/6D3 (NCL-DYS1/dystrophin rod domain), Calp3d/2C4 (NLC-CALP-2C4/calpain 3 exon 1), and ad1/20A6 (NCL-α-SARC/α-sarcoglycan) monoclonal antibodies (all from Novocastra).

Mutations were conventionally identified [19]. Deletions and duplications were detected by quantitative fluorescent-PCR using genomic DNA, and all other types of mutations were detected either by sequencing of all DMD gene exons and exon-intron junctions, or by systematic analysis of muscle dystrophin mRNA, using RT-PCR and sequencing of 14 amplified overlapping fragments. Abnormalities were substantiated by segregation analysis [19]. Altered transcripts were inferred from each mutation [20]. The four internal promoters of the dystrophin gene give rise to several transcripts. Splicing between promoter-specific first exons and exons 30, 45, 56, 63 generate respectively the dystrophin isoforms Dp260, Dp140, DP116, and Dp71. Mutations before exon 30 only affect full length dystrophin. In addition to it, the other isoforms are successively affected, as the mutation progresses after exon 29 towards the C terminus.

### Statistical Analyses

To detect correlations between clinical variables and homogenous sub-groups of patients, we used non-linear Categorical Principal Component Analysis (CatPCA) [21], followed by Hierarchical Cluster Analysis (HCA) [22]. CatPCA benefits from optimal scaling, handles together nominal (*e.g.* initial symptoms), ordinal (*e.g.* cognitive status), and interval (*e.g.* age at ambulation loss) variables, and is suitable for data recorded with uncertain units (*e.g.* MMT scores) [21]. Analysis was performed with SPSS 11.0 software. Original variables were reduced to 2 principal components with Cronbach's α≥0.70 for each, allowing plotting of cases in a two dimensional space. Clusters were determined using Statgraphics Plus 5.0. Their stability was tested by 20 randomisations, each one providing a sample containing half of patients, and by repeating HCA on each sample; cases that did not remain in the same cluster on two HCA were considered unclassifiable [21]. To identify early predictive indicators of different outcome patterns, we used a comprehensive set of diagnostic accuracy tests (sensitivity, specificity, kappa, accuracy, positive and negative predictive likelihood values), as previously recommended [23]. These tests were calculated using the Diagnostic Effectiveness module of SISA [24]. Comparisons in figures indicate differences between clusters established by multiple range post-test with (p<0.05), following a significant Kruskal-Wallis test (Statgraphics 5.0 Plus). Two-sided p-values are reported, 95% confidence intervals of means or proportions, or box-and-whisker plots (10^th^, 25^th^, 50^th^, 75^th^, 90^th^ percentiles) represent data scatter.

## Results

### Global population characteristics (Table 1)

Follow-up of the 75 patients from series 1 ranged from 4.0 to 18 yrs (mean 10.2). Most patients (n = 41) were older than 16 at end-point. Clinical characteristics are listed in table 1. Briefly, 56% of patients had delayed walking (>18 mo), 56% had never been able to run, and 31% to climb stairs without support. Ability to rise from floor was lost at 8.3 yrs, and to elevate arms at 13.7 yrs. All patients had scoliosis, diagnosed from age 9 to 16. Lower limb tenotomies were performed at 9.6 yrs (n = 42), and spinal arthrodesis at 13.2 yrs (n = 53). Patients usually had normal respiratory function until 10 yrs. Cardiac failure was diagnosed in 30% of patients at age 12, 7 died from heart failure (2 before 15 yrs, 5 after 20 yrs).

### Identification and characterisation of clusters

**Figure 1:**
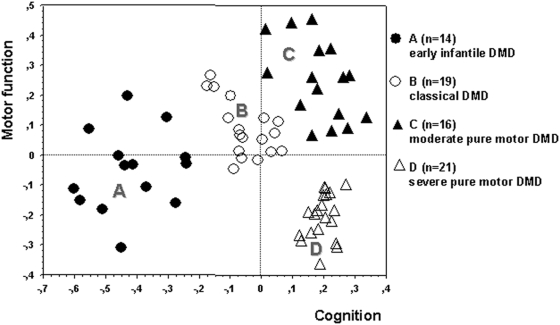
The 4-cluster solution. DMD patients were assigned to 4 clusters in the final hierarchical cluster analysis (linkage: Ward's method, metric: squared Euclidean distance) using CatPCA-derived XY coordinates in a plane defined by the cognition and motor function principal components (axes) (Cronbach's α: 0.859 and 0.721, respectively). Cognition was always altered to various extent in patients with early infantile DMD (A) and classical DMD (B), but was preserved in patients with moderate (C) and severe (D) pure motor DMD who differed markedly in motor function impairment. Overall, cluster A patients were most severely affected.

CatPCA identified cognitive and motor parameters as the main contributors to the structure of DMD symptoms, which could be adequately described by two independent principal components (figure 1). Six variables contributed to a “cognition” axis, including items describing intelligence and education and also age and symptoms at disease onset, and 4 to a “motor function” axis, including 3 items describing lower limb function plus “delay of diagnosis” which was longer in less severely myopathic patients (table 2).

**Table 2:** Variables' contributions to the two principal components (Cronbach's α, a measure by default for the internal consistency of categorical principal components in the SPSS software, is 0.891 and 0.721), absolute values above 0.500 were retained to define the principal components.

Variable	Principal component	
	1 Cognition	2 Motor function
Cognitive status	**0.891**	−0.095
Maximal education level	**0.884**	−0.109
Education delay	**0.840**	−0.376
Education type of establishment	**−0.814**	−0.020
Age at initial symptoms	**0.725**	0.132
Initial symptoms	**0.710**	−0.353
Age at becoming chair-ridden	0.226	**0.857**
Age at first contractures in lower limbs	0.298	**0.759**
Age at loss of ambulation	−0.131	**0.707**
Delay of diagnosis	0.442	**0.697**

**Figure 2:**
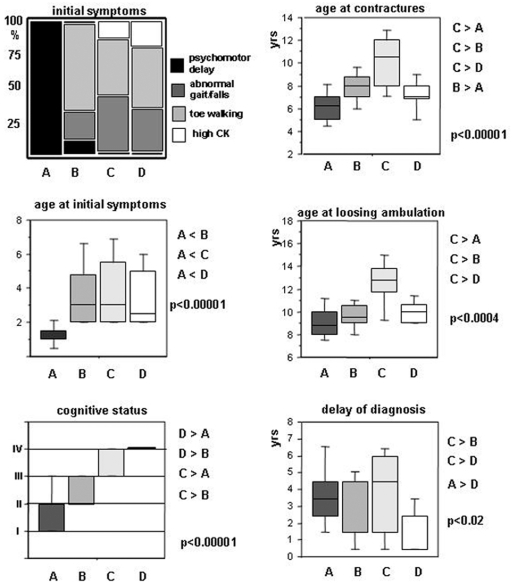
Variables constituting the “cognition” (left) and the “motor function” (right) principal components. Group A patients manifested early in life by psychomotor delay. “Cognitive status” measures increased from cluster A to D (the following classification was used: (I) severely mentally retarded; (II) mildly mentally retarded; (III) borderline; (IV) normal). This was also supported by an identical increase of “maximal education level” and an inversely symmetrical decrease of “school delay” (p<0.00001 for both, data not shown). Accordingly, 86% of D patients attended an ordinary educational establishment *vs.* 38% of C, 26% of B and 21% of A patients (all p<0.007 by Fischer exact test). A and C patients had values at each extremity of the motor function spectrum, also comprising the age of becoming “wheelchair-ridden” (p<0.00001, not shown). “Delay of diagnosis” contributed to the integrity of the second principal component and, notably, to the delineation of cluster D (indicated p values are obtained by Kruskal-Wallis test; clusters were compared by a multiple range post-test with p<0.05).

**Figure 3:**
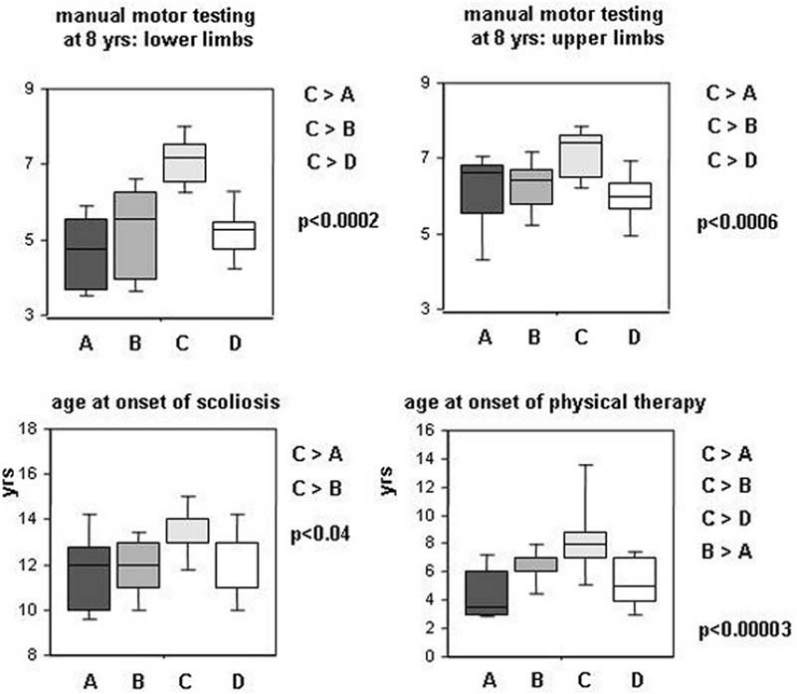
Muscle strength variables not used to constitute the model. C patients had better muscle strength than A, B, or D patients and a retarded onset of scoliosis. These motor parameters, not used in the multivariate analysis, support the validity of the model. “Age at onset of physical therapy” trailed behind the evolution of muscle strength and motor function: no clear benefit from therapy could be demonstrated in this series (indicated p values are obtained by Kruskal-Wallis test; clusters were compared by a multiple range post-test with p<0.05).

**Figure 4:**
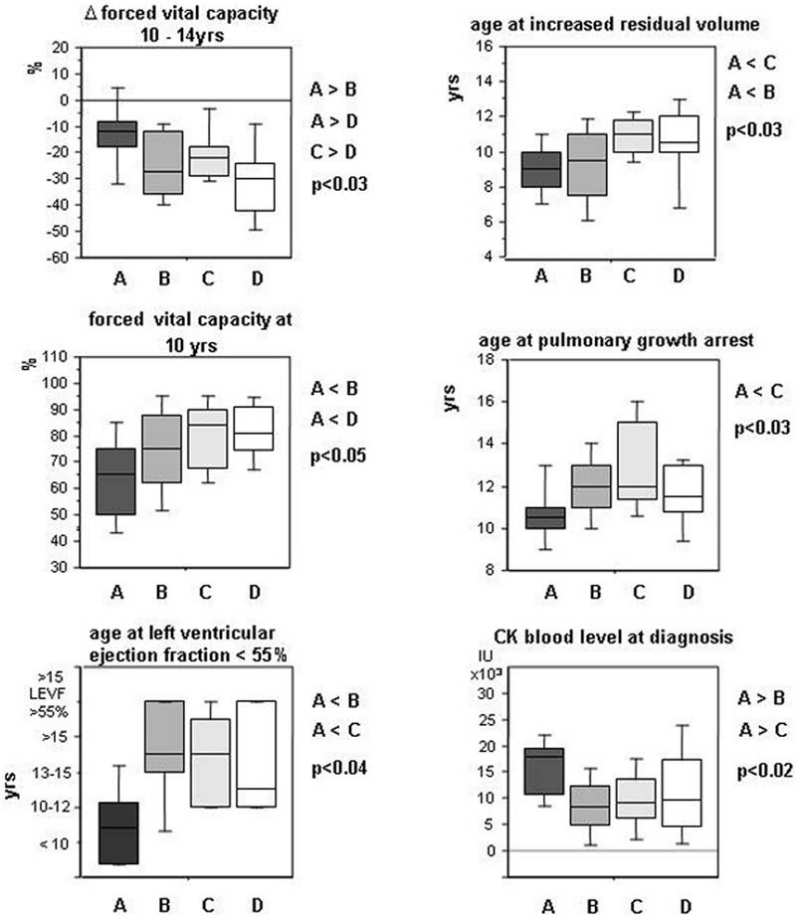
Pulmonary and cardiac function variables not used to constitute the model. Group A patients had poorest respiratory and myocardial functions and highest serum CK levels at diagnosis. These variables, not used in the multivariate model, also corroborate its predictive relevance (indicated p values are obtained by Kruskal-Wallis test; clusters were compared by a multiple range post-test with p<0.05).

HCA yielded an optimal 4-cluster solution. Cluster stability testing detected no more than 5/75 unclassifiable cases to be excluded (7%). The final solution consisted of 4 clusters (A: n = 14, B: n = 19, C: n = 16, D: n = 21). No statistically significant differences between clusters could be demonstrated for duration of follow-up: A 10.1 (95% CI: 8.0 to 12.2 yrs), B 9.3 (95% CI: 7.3 to 11.4 yrs), C 9.9 (95% CI: 7.8 to 12.0 yrs), D 11.1 (95% CI: 9.6 to 12.6 yrs), p = 0.47, Kruskal-Wallis test. Clusters were characterised by highly distinctive phenotype and outcome on both cognition and motor function axes (figure 2, left and right columns, respectively). Importantly, clinical variables not used to constitute the model also showed significant differences between clusters (figures 3,4), thus providing basis for the recognition of 4 distinctive DMD subsets: group A (early infantile DMD, 20%) very poor intellectual and motor outcomes; group B (classical DMD, 28%) intermediate intellectual and poor motor outcomes; group C (moderate pure motor DMD, 22%) normal intelligence and delayed motor impairment; and D (severe pure motor DMD, 30%) normal intelligence and poor motor outcome.

Group A was characterised by both precocity and severity of symptoms. Initial symptoms appeared at 1.3 yrs (*vs.* ≥3.3 yrs in other groups) and always consisted of psychomotor delay. Patients had the poorest mental status (29% IQ<50; 57% IQ 50-70) and global outcome, as assessed by generally lower cognitive, motor, cardiac and respiratory (figure 4) indices than other groups; 13/14 (93%, 95% CI: 72 to 99%) of these patients were never able to run versus 48% (95% CI: 48 to 62%) in other groups (p<0.005). They had the highest CK levels at diagnosis (figure 4); 10/14 (72%° had cardiomyopathy before 12 yrs, and 13/14 (90%) at 15 yrs.

Group B patients had clinical features and severity similar to the mean values of the overall DMD population, thus corresponding to what is considered the common clinical DMD profile in the literature. They had poor motor outcome (ambulation loss at 9.6 yrs) and mildly impaired mental status; only 3/19 (16%) of patients with cardiac and respiratory dysfunctions before 12 yrs. Group B showed significantly better global outcome than group A, worse mental status than groups C and D, and worse motor status than group C.

Group C patients had the best motor outcome and almost normal cognition. Ambulation loss occurred at 12.5 yrs, and MMT at 8 and 10 yrs was the best measured among DMD patients in both the lower and the upper limbs; contractures and scoliosis onsets were delayed (figure 4); Since DMD diagnosis was delayed in these patients (age 7.2 yrs), physiotherapy was applied later thus excluding better outcome linked to rehabilitation. BMI was intriguingly different, 11/16 (69%) (95% CI: 41 to 89%) of patients being thin *vs.* 18/54 (33%) (95% CI: 21 to 48%) in other groups (p<0.02).

Group D patients had a poor motor outcome similar to group B, but strictly normal cognition. Ambulation loss occurred at 10 yrs. Patients had strictly normal cognitive status and an educational level similar to this of group C. However, 86% of D patients attended an ordinary educational establishment *vs.* 38% of C, 26% of B and 21% of A patients (all p<0.007 by Fischer exact test).

### Genotype-phenotype correlation

Mutation was identified in 67 patients, including 77% deletions, 19% punctual mutations, and 4% duplications. Mutations are predicted to mainly affect expression of transcripts corresponding to DP427 isoform (mutation in the region of exons 1–29, n = 24), DP427+DP260 (exons 30–44, n = 18), and DP427+DP260+DP140 (exons 45–55, n = 20). Additional involvement of DP116 (exons 56–62), and of DP116+DP71 (after exon 63) occurred in 2 and 3 patients, respectively.

**Figure 5:**
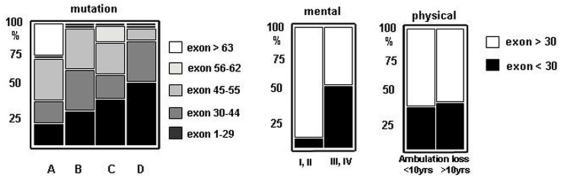
Genotype/phenotype correlations. Proportion of patients with a mutation upstream to exon 30 steadily increased from group A to D. This ascent correlated with spared cognition (mental status: p<0.0003) but not with motor function (age at ambulation loss: NS) (Fisher's exact test). Expectedly, the 3 patients with mutation after exon 63 affecting the brain specific DP71 transcript were classified in group A.

Clinical classification of patients partially correlated with the gene affected region (figure 5), as mutations before exon 30 were found in increasing numbers from group A to D: 18% (A); 29% (B); 40% (C), and 55% (D). Further, we separately explored genotype impact on cognition and motor function. We found that frequency of mutations before exon 30 correlated well with IQ (IQ>71: OR 7.7, 95%CI: 1.6 to 20.4, p<0.003) and retinogram (normal retinogram: OR 9.7, p<0.007), whereas no correlation with any motor parameter could be demonstrated (e.g. age of ambulation loss >10 yrs: OR 1.2, 95%CI: 0.4 to 3.4, p = 0.44). As expected, the 3 patients with mutation after exon 63 affecting the brain specific DP71 transcript had severe mental retardation [20] and were classified in group A.

### Selective indicators for patient classification

Then, we examined if a reduced set of criteria could be sufficient to classify patients. Using diagnostic accuracy tests, we searched for simple and widely available indicators allowing discrimination of patients from group A *vs.* B+C+D, group C *vs.* A+B+D, and group B *vs.* D. Results are listed in tables 3 and 4. Consistent with the precocity of their psychomotor symptoms, patients classified in group A were reliably identified by “psychomotor delay as first symptom” (sensitivity: 0.93, specificity: 0.95). In the same way, group C patients, whose motor handicap less severe had and occurred later in life, could be identified by “loss of ambulation >11 yrs” (sensibility 0.78, specificity 0.96). These two indicators appeared as suitable and simple surrogate criteria for recognition of group A and group C patients, respectively. Combinations of criteria also gave good results. Combination of “clinical onset <2 yrs” with “mental retardation” (severe or mild) assigned patients to group A (sensitivity 0.93, specificity 0.98) with even higher positive predictive value than the criterion “psychomotor delay as first manifestation” (PPV: 0.93 *vs.* 0.81). Combination of “lower limb MMT score>6 at 8 yrs” with “normal or borderline cognitive status” most reliably assigned patients to group C (sensitivity: 1, specificity: 0.94). Compared to the criterion “loss of ambulation >11 yrs”, combined criteria showed higher accuracy and offered the advantage of being usable at a much younger age. Combined criteria allowed exclusion of group A and C patients from the series. In the remaining population, IQ categorically segregated group D from group B patients. Notably, predictive combinations reliably identified patients classified by surrogate indicators as early infantile DMD (table 3) and moderate pure motor DMD (table 4).

**Table 3:** Simplified indicators of congenital DMD (cluster A) derived from series 1.

	Sensitivity	Specificity	Accuracy	Kappa	PPV**	NPV**
Psychomotor delay as 1^st^ symptom→congenital DMD defined by cluster analysis*	0.93	0.95	0.94	0.83	0.81	0.98
	0.64–1.00	0.84–0.99	0.85–0.98	0.60–1.00	0.57–0.93	0.91–1.00
Age initial symptom <2 yrs+cognitive status I, II***→congenital DMD defined by cluster analysis*	0.93	0.98	0.96	0.91	0.93	0.98
	0.64–1.00	0.89–1.00	0.84–0.99	0.68–1.00	0.68–0.98	0.91–1.00
Age initial symptom <2 yrs+cognitive status I, II→congenital DMD defined by surrogate criterion (psychomotor delay as 1^st^ symptom)*	0.88	1.00	0.97	0.92	1.00	0.94
	0.64–0.98	0.92–1.00	0.89–1.00	0.68–1.00	0.78–1.00	0.81–0.99

* Yate's Chi square: all p = 0.00001, exact 95% confidence limits.

** PPV and NPV, positive or negative predictive value.

*** I: severe mental retardation, II: mild mental retardation (see methods).

**Table 4:** Simplified indicators of moderate pure motor DMD (cluster C) derived from series 1.

	Sensitivity	Specificity	Accuracy	Kappa	PPV**	NPV**
Ambulation loss >11 yrs→moderate pure motor DMD defined by cluster analysis*	0.78	0.96	0.91	0.77	0.88	0.93
	0.59–0.97	0.91–1.00	0.85–0.98	0.53–1.00	0.71–1.00	0.80–1.00
MMT>6 at 8 yrs plus+cognitive status III, IV→moderate pure motor DMD defined by cluster analysis*	1	0.94	0.96	0.88	0.83	1.00
	0.72–0.99	0.80–0.99	0.84–0.99	0.59–1.00	0.55–0.95	0.80–0.99
MMT>6 at 8 yrs plus+cognitive status III, IV→moderate pure motor DMD defined by surrogate criterion (ambulation loss >11 yrs)*	0.83	0.94	0.91	0.78	0.83	0.94
	0.51–0.97	0.79–0.99	0.78–0.97	0.49–1.00	0.55–0.95	0.81–0.99

* Yate's Chi square: all p = 0.00001, exact 95% confidence limits.

** PPV and NPV, positive or negative predictive value.

*** III borderline normal, IV normal (see methods).

To test the robustness of these predictive criteria, we examined an additional set of 34 unselected DMD patients aged more than 12 (series 2). Files contained data required to retest group A and C patients (age of onset with type of initial symptoms, n = 32; and MMT at 8 yrs with mental status evaluation, n = 34), but were not complete enough to classify patients by the multivariate model. Surrogate criteria, however, allowed satisfactory recognition of patients with clinically evident early infantile DMD (“psychomotor delay as first manifestation”, n = 8) and with moderate pure motor DMD (identified *a posteriori* by “loss of ambulation >11 yrs”, n = 9). Data for major motor parameters such as MMT of lower limbs, ages at loosing ambulation or at onset of scoliosis, and of becoming wheel-chair-ridden were very similar for moderate pure motor DMD patients identified by multivariate analysis (series 1) or by surrogate criteria (series 2) (not shown). As shown in table 5, early predictive criteria in combination assigned patients in these two subgroups, with similarly good efficiency in series 1 and 2.

**Table 5:** Simplified indicators applied to congenital and to moderate pure motor DMD in series 2.

	Sensitivity	Specificity	Accuracy	Kappa	PPV**	NPV**
Age initial symptom <2 yrs+cognitive status I, II***→psychomotor delay as 1^st^ symptom*	0.86	0.96	0.94	0.86	0.86	0.96
	0.47–0.97	0.80–0.99	0.77–0.99	0.47–1.00	0.47–0.97	0.80–0.99
MMT>6 at 8 yrs plus+cognitive status III, IV***→ambulation loss >11 yrs*	1.00	0.92	0.94	0.86	0.82	0.94
	0.63–0.99	0.73–0.99	0.79–0.99	0.53–1.00	0.52–0.95	0.86–1.00

* Yate's Chi square: all p = 0.00001, exact 95% confidence limits.

** PPV and NPV, positive or negative predictive value.

*** I severe mental retardation, II mild mental retardation, III borderline normal, IV normal (see methods).

## Discussion

In this study, we substantiated DMD's clinical heterogeneity and identified 4 subsets of patients with different cognitive and motor outcomes. Despite rough correlation of cognitive impairment with the mutated region in the dystrophin gene, and of low BMI with better motor outcome, neither genotyping nor nutritional status served to identify DMD subsets, whereas age and type of symptoms at onset, muscle strength at 8 yrs, and IQ, did.

Unlike previous studies conducted on the whole spectrum of dystrophinopathies [9], [10], we stringently included patients without any residual dystrophin at Western blot. Mutation could be identified in 89% of patients, a satisfactory prevalence for DMD series. Global characteristics of our DMD population were similar to those previously reported in terms of ages at diagnosis (5.2 *vs.* 5.3 yrs), first walking (16 *vs.* 20 mo), first symptoms (3 *vs.* 2.4 yrs), and chair-ridden (10.2 *vs.* 9.5 yrs) [8].

Admittedly, our study has the drawbacks of any retrospective clinical investigation, such as variable follow-up duration which occasionally resulted in missing data for delayed events in the youngest patients (*e.g.* spinal arthrodesis or cardiomyopathy). A long-term follow-up (>10 yrs) prospective study on the global clinical variables of DMD, however, is unlikely to be undertaken.

A strong point of the study is that patients were followed by the same medical team, collecting data on a systematic basis twice a year and maintaining homogeneous practices along the entire follow-up. For example, none of the patients had received steroids.

Multivariate classification approaches can be applied to small cohorts [25], [26]. They were used for example to subclassify spinal muscular atrophy patients (n = 102) into 3 severity types [27] or to delineate specific epilepsy syndromes (n = 72) for therapeutic purposes [22]. The power of such approaches reside in their ability to uncover, without *a priori* hypotheses, variables that participate to data structure [28]. These variables may seem poorly relevant when considered individually, e.g. “type of educational establishment” and “delay of diagnosis” strongly contributed to the stable 4-cluster solution.

The 4 DMD subsets mainly differed by cognitive and motor involvement.

Group A included 20% of patients with early infantile form of DMD, somewhat similar to that previously described as congenital DMD [29]. These patients came early to medical attention (1.2 yrs) because of psychomotor delay affecting speaking more than walking. Severe (1/3) to moderate (2/3) mental retardation was constant. Despite early onset, diagnosis was delayed considerably and was performed usually when motor deficit had become obvious. They had the poorest motor, respiratory and cardiac outcomes, *i.e.* the most severe striated muscle involvement. For example, 2/3 of them had cardiac dysfunction before age 12. Accordingly, the highest CK levels were found in this group. Finally, patients with psychomotor delay as the initial manifestation, with marked hyperCKaemia, and who have never been able run, form an easily recognizable subset of severe DMD.

Group B designated as classical form of DMD, included 28% of patients with clinical features and functional parameters similar to the overall DMD population. Despite poor motor outcome and constant learning difficulties, these patients could not be easily recognised due to lack of specific or salient clinical characteristics.

Group C (moderate pure motor DMD) included 22% of patients, sharply contrasting with the others because of a better muscular status. As a corollary, both DMD diagnosis (7.2 yrs) and rehabilitation onset were delayed in these patients. Intriguingly, most of them attended motor handicapped schools, and were thin according to BMI (69% *vs.* 31% in other groups, p<0.03). Why nutritional status in DMD is highly variable remains unclear, but low BMI in group C patients supports the view that limited burden of the weakened muscles may be an advantage in DMD [30].

Group D patients (severe pure motor DMD) included 30% of patients with sharp contrast between severe motor involvements and perfectly spared cognitive functions. Complementary Western blot analyses were systematically conducted to explain discrepancy of muscle involvement in groups C and D patients. They failed to detect different levels of relevant muscle proteins including dystroglycans, sarcoglycans, dysferlin, and calpain (data not shown).

There were inter-group genotypic variations as mutations before exon 30 increased in numbers from group A to D. Location of the mutation selectively influenced cognitive but not motor outcome. When cognition was evaluated separately, correlations remained incomplete, except for rare mutations occurring after exon 63 that were exclusively found in group A and associated with severe mental retardation [20]. Lack of correlation with motor outcome suggests impact of other factors than the mutation itself on severity of the myopathy [31]. Whether these factors implicate genetic components remains to be established [4]. As genotyping appeared nearly useless to predict motor outcome, we searched for robust clinical indicators allowing early classification of almost any DMD patient. Group A patients could be easily recognised from infancy by psychomotor delay occurring as initial symptom before 2 yrs. Moreover, group C patients, which clinical status worsens more slowly than usual in DMD, could be reliably recognised from 8 yrs of age by combination of “lower limb MMT score >6” with “normal or borderline mental status”. We were unable to use earlier indicators for this group, *e.g.* MMT at 6 yrs, because many group C patients were not diagnosed at this age, due to less severe myopathy and longer diagnosis delay (usually >4 yrs). Once A and C patients were excluded, group D patients could be easily distinguished from group B patients on the grounds of their constantly normal IQ.

With regard to therapeutics, it would be interesting to examine if “good responders” to steroids belong to a given sub-phenotype of DMD. More generally, trials, which are in danger of being inconclusive due to lack of precise knowledge on DMD's natural history [7], would strongly benefit from accurate selection of clinically homogeneous patient subsets. The simple combinations of robust predictive criteria identified in this study seem appropriate for this purpose.
